# Evaluation of S100A12 and Apo-A1 plasma level potency in untreated new relapsing–remitting multiple sclerosis patients and their family members

**DOI:** 10.1038/s41598-022-06322-4

**Published:** 2022-02-09

**Authors:** Mahsa Samangooei, Mojtaba Farjam, Masoud Etemadifar, Atefeh Taheri, Mohammad Hassan Meshkibaf, Bahram Movahedi, Zahra Niknam, Saam Noroozi

**Affiliations:** 1 Department of Clinical Biochemistry, Fasa University of Medical Sciences, Fasa, Iran; 2 Noncommunicable Diseases Research Center, Fasa University of Medical Sciences, Fasa, Iran; 3 Department of Functional Neurosurgery, Isfahan University of Medical Sciences, Isfahan, Iran; 4 Shiraz, Iran

**Keywords:** Neurological disorders, Neurogenesis

## Abstract

Multiple sclerosis is an inflammatory disease of the spinal cord and brain. Receptor for advanced glycation end products and Apolipoprotein A1 (Apo-AI) have been recommended to have a pathogenic role in the neuroinflammatory disorder as multiple sclerosis. The purpose of this research was to measure the plasma levels of S100A12 and Apo-A1 in the first-degree family of relapsing–remitting multiple sclerosis (RRMS) patients. Plasma levels of S100A12 & Apo-A1 were evaluated via enzyme-linked immunosorbent assay in the thirty-five new cases of untreated patients with deterministic RRMS according to the McDonald criteria, twenty-four healthy controls, and twenty-six first-degree members of untreated RRMS patients (called them as high-risk group). The main findings of this study were as follows: the plasma level of S100A12 was significantly lower in the new cases of untreated RRMS (*P* ≤ 0.05; 0.045) and high-risk (*P* ≤ 0.05; 0.001) groups. Although the plasma protein level of Apo-A1 was reduced significantly in the high-risk group (*P* < 0.05, *P* = 0.003) as compared to the healthy control group, there was no significant difference in the untreated RRMS patients (*P* = 0.379). The plasma level of vitamin D3 in both RRMS patients and high-risk groups displayed significance reduction, although, there was no significant association between vitamin D and S100A12 & Apo-A1 levels. Given the role of S100A12 and Apo-A1 in the inflammatory process performed in the first-degree family members of the RRMS patients, which revealed a significant decrease in this group, we concluded that they can be considered as one of the contributing factors in the pathogenesis of MS, though more research is needed before assuming them as predictive biomarkers.

## Introduction

Multiple sclerosis (MS) is a neuroinflammatory autoimmune disorder of the central nervous system (CNS)^1,2^, gradually damages the myelin sheath and forms plaques on the surface of neurons^3^. The onset of the disease commonly occurs between the ages of 20 and 50^4^. MS affects around 2.5 million people worldwide^5^. In general, the prevalence of MS is increasing in Iran^6^. The proportion of women to men in Iran has risen from 2 in 2002 to 3.22 in 2007^7^. The prevalence of MS in Iran varies geographically from 5.3 to 74.28 per 100,000 people. The origins of this variety are still unclear. Ethnicities, cultures and climates all have a role^8^. While the exact aetiology of MS is unknown, scientists believe that a number of factors may be involved in MS disorder^9^. MS susceptibility is inherited, according to genetic research. Studies on families and twins back up the hypothesis that the disease is genetic^10^. Despite the fact that MS does not have a straightforward Mendelian inheritance pattern, the risk of getting the disease increases as the percentage of persons who are affected rises^11^. Individual relatives, especially first-degree relatives in Canadian families, have a 20–50 times higher chance of having MS than other members of society, according to genetic studies of the population, families, and twins^12^. Roughly 20% of patients with MS have at least one relatively impacted^13^. Incidence of MS is nearly two times greater among women than in men, although it affects males faster and with more severe symptoms^14^.

When the immune system is confronted with a threat, it releases molecules termed damage-associated molecular pattern protein (DAMPS), which first introduced in 2004 by Seong and Matzinger^15^. S100/calgranulin protein is a low molecular weight protein (9–13 kd) binds to Ca^+2^, It is composed of several members, including S100A8, S100A9, and S100A12^16^. S100A12’s inflammatory functions include chemotactic and intrinsic signaling which induce a variety of cytokines. Inflammatory responses are enhances when S100A12 binds to RAGE^17^. In chronic inflammation conditions such as rheumatoid arthritis, bowel disorder and cystic fibrosis, S100A12 plays a key role in local inflammatory reactions. RAGE binds some proinflammatory ligands, like members of the S100/calgranulin family, and is though S100A12 promote proinflammatory function by binding and activating RAGE^18^. Apolipoprotein A1 (Apo-AI), the most abundant component of high-density lipoprotein (HDL)^19^, has function as anti-inflammatory molecule in both infected and non-infectious circumstances, although its role in the pathogenesis of MS has not been fully revealed^20^. Apo-A1 can use as novel therapeutic methods in inflammatory disorders like rheumatoid arthritis, multiple sclerosis, systemic lupus erythematosus, and atherosclerosis^21^. Apo-A1 as a clinical marker has recently been demonstrated to respond to interferon-beta therapy in MS patients. Patients treated with IFN 23 had considerably decreased plasma levels of Apo-A1 at the end of the first year of treatment, according to studies^22^. In the CNS, Apo-A1 may be used as a marker of neural degeneration in patients with Alzheimer’s disease (AD), Parkinson’s disease and MS^23^. Few studies examined the effect of Apo-AI on RRMS patients. Around 30 years ago, it was proposed that vitamin D deficiency could be a risk factor for MS. The role of vitamin D in the pathophysiology and clinical course of MS has become a focus of intense research, raising many questions and controversies among physicians who treat MS patients. Sunlight exposure, the usage of vitamin D supplements, and greater levels of vitamin D in the blood were linked to a lower risk of MS incidence^24^. Low vitamin D levels, particularly values less than 10 ng/ml, may worsen autoimmune diseases such as MS^25^.

As a result, this is the first study to look at the link between Apo-AI and S100A12 plasma levels in MS patients' family members, in this research called theme as a high-risk group. Although it is generally accepted that inflammation plays a major role in the pathogenesis of multiple sclerosis, our knowledge about the inflammatory regulation and mechanism, as well as the recognition of the several mediators are not complete. The discovery of novel biomarkers throughout this process has the potential to improve the efficacy of employing them to treat this condition and provide a new therapeutic target.

### Results

The demographic information of the patients is shown in Table 1. Table 2 summarizes the plasma concentrations of S100A12 and Apo-A1.

**Table 1:** Baseline demographic and clinical characteristics of participants of study.

Groups	Healthy controls	Untreated RRMS Patients	High-risk
NO	24	35	26
Age: (Mean ± SD)	29.33 ± 8.17	30.14 ± 9.09	32.08 ± 14.44
*p*-value		n.s	n.s
Sex: (F/M)	19:5	26:9	16:10
EDSS	–	1.3 ± 0.9	–
Smoking: (F/M)	0:1	2:4	1:5
Height: (m)	1.61 ± 5.64	1.65 ± 5.94	1.62 ± 8.95
*P*-value		n.s	n.s
Weight: (Kg)	64.42 ± 8.11	60.63 ± 5.16	59.50 ± 8.81
*P*-value		< 0.001	< 0.001
BMI	24.65 ± 2.55	22.33 ± 2.11	22.42 ± 2.46
*P*-value		0.001	0.006
CRP: (mg/dl)	1.07 ± 0.18	3.84 ± 1.23	1.19 ± 0.57
*P*-value		< 0.001	0.03
NLR	1.22 ± 1.03	2.73 ± 0.98	2.03 ± 1.16
*P*-value		< 0.001	< 0.001
Other neurological or Inflammatory Disease	–	–	–

*EDSS* Expanded disability status scale with ranging 0–10, *MS* multiple sclerosis, *RRMS* relapsing–remitting multiple sclerosis, *CRP* C-reactive protein, *NLR* neutrophil–lymphocyte ratio.

**Table 2:** The mean concentration of S100A12 and Apo-A1 plasma levels quantified by ELISA.

Group	No	S100A12 (pg/mL)	*P*-value	Apo-A1 (ng/mL)	*P*-value
Healthy controls	24	42.58		205.88	
Untreated RRMS Patients	35	36.78	*P* ≤ 0.05	171.71	n.s
High-risk	26	15.97	*P* ≤ 0.05	111.78	*P* ≤ 0.05

### S100A12 plasma levels in untreated RRMS and high-risk

**Figure 1:**
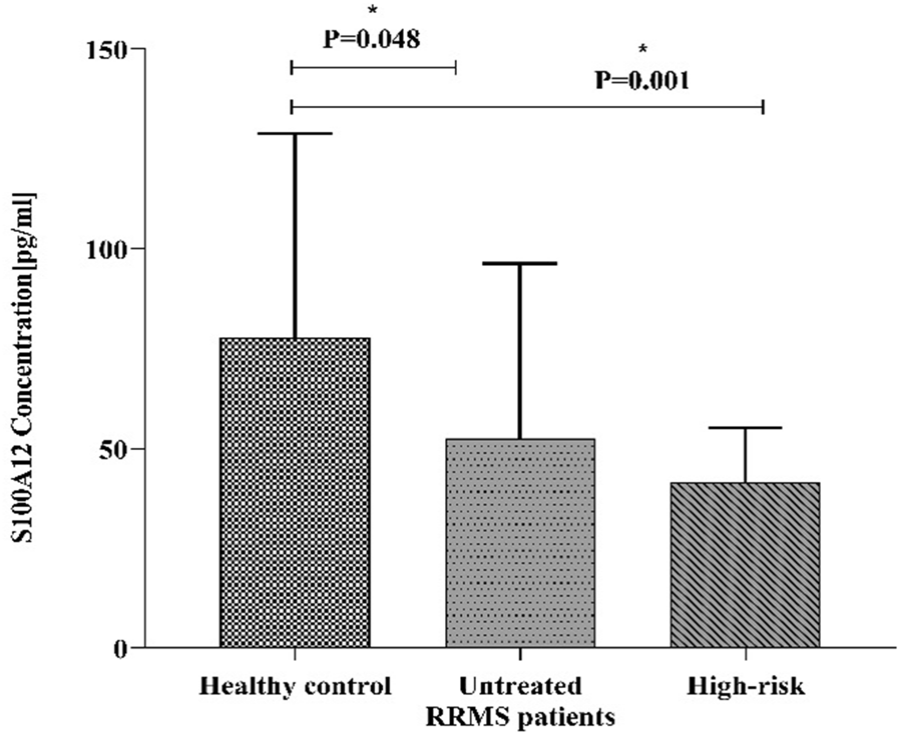
Represented the S100A12 concentration by mean ± SD in the different groups in three boxes. According to this chart, the concentration of S100A12 decreased significantly in two untreated RRMS and high-risk groups compared to the healthy control group (*P* < 0.05).

According to the findings of this investigation, plasma levels of S100A12 were significantly lower in two groups: untreated RRMS and high-risk (Family members of untreated RRMS patients: their sibling and parents), as compared to a healthy control group. The Concentration of S100A12 in the untreated RRMS group was (mean ± SD = 36.781 ± 48.182 pg/ml), in healthy control (mean ± SD = 42.586 ± 43.641 pg/ml) and in the high-risk group (mean ± SD =15.979 ± 13.163 pg/ml). S100A12 plasma level decreased significantly in untreated RRMS (ANOVA, *P* ≤ 0.05; 0.045) and high-risk (ANOVA, *P* ≤ 0.05; 0.001) as compared with healthy control (Fig. 1).

### Apo-A1 plasma levels in untreated RRMS and high-risk

**Figure 2:**
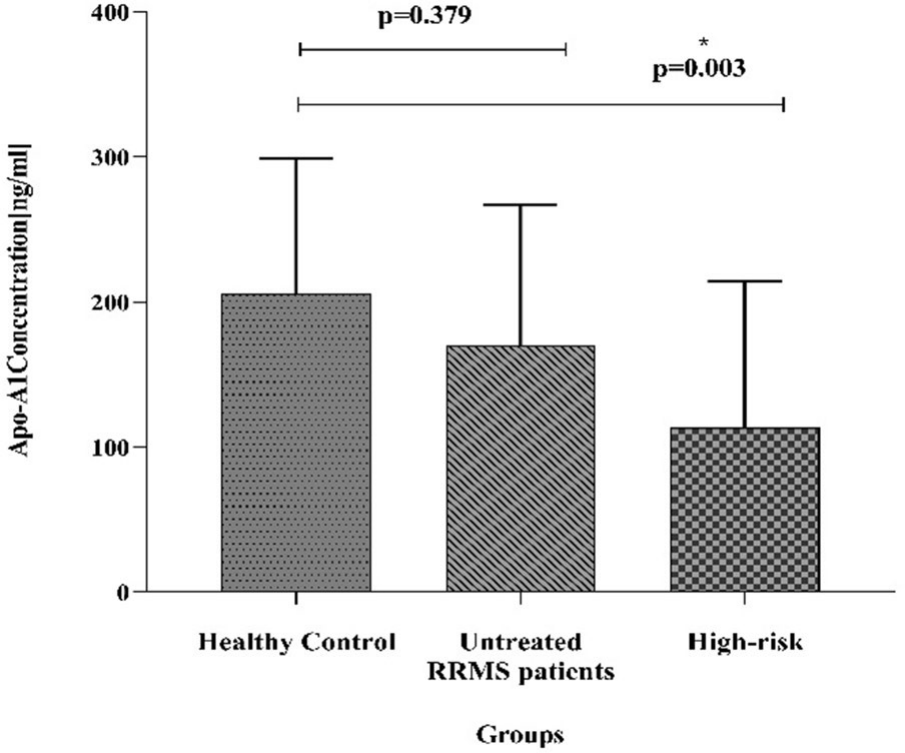
The Apo-A1 plasma level displayed in the healthy controls, untreated new case RRMS, and high-risk groups by mean ± SD. The mean plasma Apo-A1 level between the control and the high-risk groups was reduced markedly (*P* = 0.003). The difference between the untreated RRMS and the healthy control groups was presented by the post-hoc test (*P* = 0.379).

The results shown, the plasma levels of Apo-A1 was significantly lower in the high-risk group than the control group (ANOVA, *P* ≤ 0.05, 0.003). Apo-A1 concentration in the new untreated RRMS group was (mean ± SD = 171.71 ± 94.39 pg/ml), in healthy control (mean ± SD = 205.88 ± 92.97 pg/ml) and in high-risk group (mean ± SD = 111.78 ± 101.92 pg/ml). Although contrary to expectations no statistically significant differences were observed in Apo-A1 levels between the HC and untreated RRMS groups (ANOVA, *P* = 0.379) (Fig. 2).

### S100A12 plasma levels and sex in in untreated RRMS and high-risk

**Figure 3:**
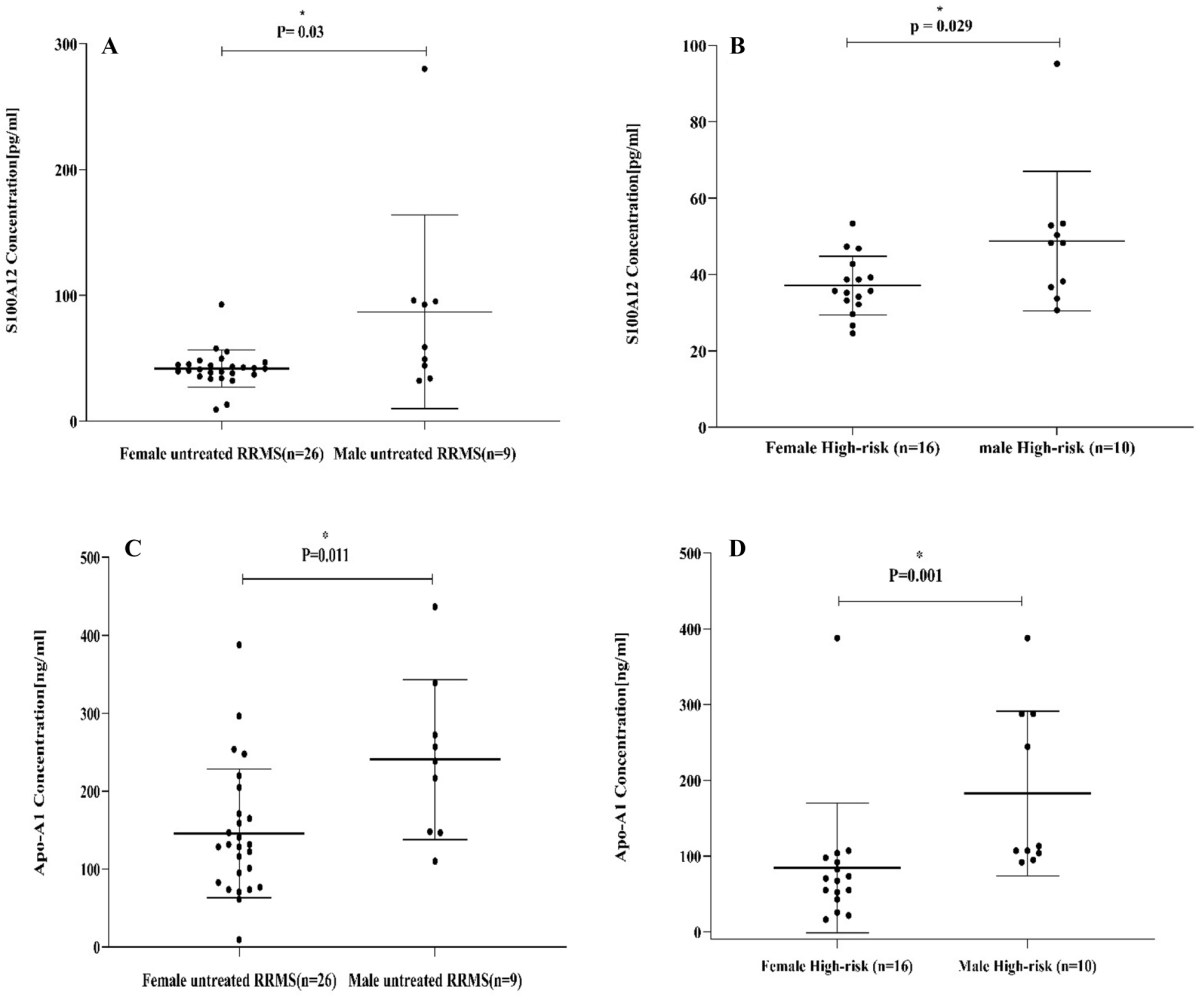
In the new cases untreated RRMS patients, S100A12 level was declined in the female more than in males (Mann–Whitney; *p* = 0.03) (**A**). S100A12 level was also reduced in the females than in the males in the high-risk group (Mann–Whitney; *P* = 0.029) (**B**). Apo-A1 levels were decreased in the females than males in both untreated RRMS patients and high-risk groups respectively (Mann–Whitney; *P* = 0.011) (**C**) (Mann–Whitney; *P* = 0.001) (**D**).

Plasma S100A12 levels were lower in females than males in untreated RRMS patients (Mann–Whitney; *P* = 0.03, Fig. 3A), also the plasma S100A12 levels were reduced in females than males in high-risk group (Mann–Whitney; *P* = 0.029, Fig. 3B).

### Apo-A1 plasma levels and sex in in untreated RRMS and high-risk

Plasma Apo-A1 levels were measured in females and males in the untreated RRMS and high-risk groups, respectively, to determine the effect of sex on Apo-AI biomarker. The Apo-A1 plasma levels were lower in the female than in the male among untreated RRMS patients (Mann–Whitney; *P* = 0.011, Fig. 3C), and the plasma Apo-A1 levels were decreased in female than in male in the high-risk group (Mann–Whitney; *p* = 0.001, Fig. 3D).

### S100A12 plasma levels and age in untreated RRMS and high-risk

**Figure 4:**
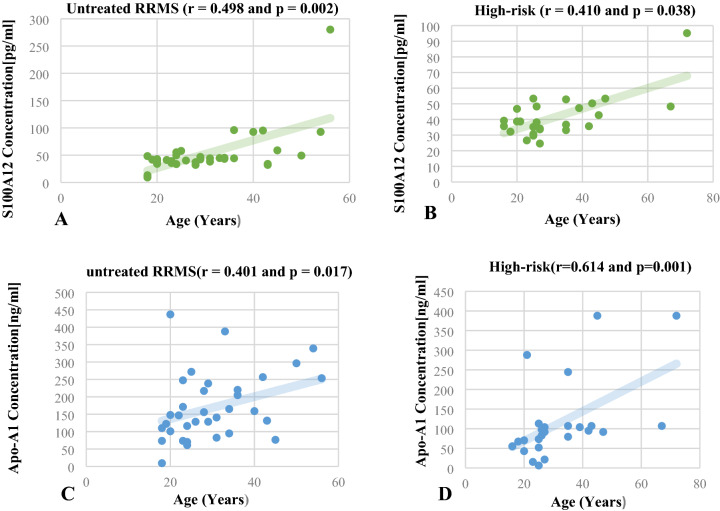
The plasma concentration of S100A12 showed a positive correlated with the age in the untreated RRMS group (Spearman; r = 0.498 and *P* = 0.002) (**A**), and in the high-risk (Spearman; r = 0.410 and *P* = 0.038) (**B**). Apo-A1 plasma level also revealed a positive correlated to the age in both untreated RRMS and in the high-risk groups (Spearman; r = 0.401 and *P* = 0.017) (**C**), (Spearman; r = 0.614 and *P* = 0.001) (**D**) respectively.

S100A12 plasma concentrations levels showed a positive correlated to age (years) in the untreated RRMS.

(Spearman; *r* = 0.498 and *P* = 0.002) and high-risk group (Spearman; *r* = 0.410 and *P* = 0.038) (Fig. 4A,B).

### Apo-A1 plasma levels and age in untreated RRMS and high-risk

Concentrations of Apo-A1 plasma showed a positive correlated to age (years) in the untreated RRMS (Spearman; r = 0.401 and *P* = 0.017) and high-risk group (Spearman; r = 0.614 and *P* = 0.001) (Fig. 4C,D).

Furthermore, there was no correlation between S100A12 and Apo-A1 plasma concentrations and EDSS (not shown). All P-values of biological, clinical gender, and age related to both biomarkers in the experimental groups are shown in Table 3.

**Table 3:** *P*-values of biological, clinical gender, and age related to both biomarkers in the experimental groups.

	Spearman’s correlation	*P*-value
**S100A12 versus sex**		
In RRMS patients		0.03
In High-risk group		0.029
**S10012 versus age**		
In RRMS patients	0.498	0.002
In High-risk group	0.410	0.038
**Apo-A1 versus sex**		
In RRMS patients		0.011
In High-risk group		0.001
**Apo-A1 versus age**		
In RRMS patients	0.401	0.017
In High-risk group	0.614	0.001

*P*-values were measured by Mann–Whitney test.

### Vitamin D plasma levels in untreated RRMS and high-risk

**Figure 5:**
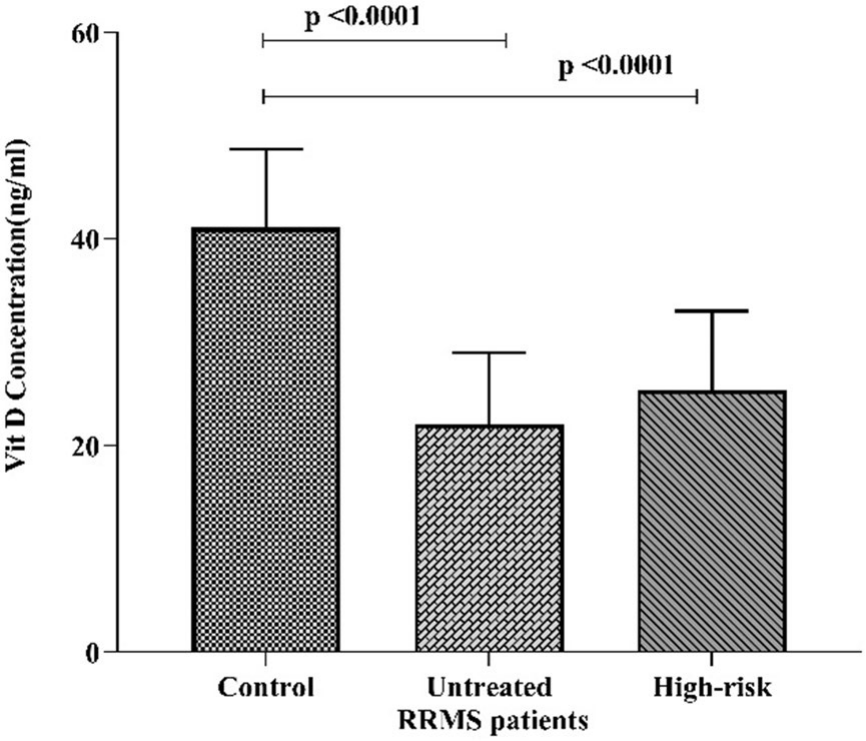
The chart represented the mean plasma level of Vit D. In both untreated RRMS and the high-risk groups the concentration of Vit D decreased markedly (Mann Whitney, *P* < 0.0001). The mean concentration of Vit D in the healthy control group (42.23 ng/ml, sufficient), in untreated RRMS (17.40 ng/ml, insufficient), and in the high-risk group was (22.58 ng/ml, insufficient).

All of cases in the study were referred to the central laboratory for evaluating vitamin D levels. Vitamin D (25 OH-D) were categorized into three groups: deficient: < 20 ng/ml, insufficient 20–30 ng/ml and sufficient 30–70 ng/ml (Table 4, Fig. 5).

**Table 4:** The mean concentration of Vit D (25OH-D) $\pm {\text{SD}}$ in the experimental group.

Group	No	Vit D (ng/ml)	*P*-value
Healthy controls	24	42.33 ± 9.21	
Untreated RRMS patients	35	17.40 ± 4.91	0.0001
High-risk	26	22.58 ± 7.41	0.0001

There was significant alteration of the plasma levels of vitamin D3 (Mann Whitney, *P* < 0.0001) in the untreated RRMS patients & high-risk group when compared to the healthy controls. The mean Vit D plasma levels in the healthy control, untreated RRMS patients and in the high-risk group was (41.08 $\pm$ 7.60 ng/ml, sufficient), (22.03 $\pm$ 6.90 ng/ml, insufficient), (25.35 $\pm$ 7.66 ng/ml, insufficient) respectively.

## Discussion

Previous research on S100A12 has revealed that it plays a role in inflammatory diseases. In 2014, Anton Glasnovi et al. reported that sRAGE decreased significantly in the CSF of MS patients, despite the fact that S100A12 expression, as measured by qPCR, did not show a significant decrease in the plasma of MS patients^26^. Another investigation by Gholamreza Asadikaram and colleagues in 2016 found that the mRNA expression levels of S100A12 were dramatically lowered in untreated new cases of RRMS^27^. Mahnoosh Rahim's study found that patients treated with interferon 28 had higher levels of S100A12 gene expression^28^.

On the other hand, investigations based on Apo-A1 biomarker in RRMS cases are few. Patients with higher Apo-A1 levels have been shown to respond better to interferon therapy, which is the most commonly used RRMS therapy^29^. Higher levels of HDL are linked to lower inflammation. ApoA-I is thought to play a role in the cognitive abilities of MS patients. It can be used to determine a patient's prognosis. The authors of the Koutsis study found that there is a link between cognitive function and the Apo-A1-75G/A promoter polymorphism, as evidenced by differential Apo-AI expression in the CSF and serum of MS patients^30^. Apo-AI may play a protective function in inflammation and autoimmune, according to several studies^31,32^. Apo-AI as a constitutive anti-inflammatory factor, may also have a role in neural regeneration^33^. The connection between blood lipids and disabilities with MRI results 34 was studied in 2011 by Weinstock-Guttman et al^34^. According to Meyers et al., plasma Apo-AI levels in progressive MS patients were lower than in stable RRMS patients and healthy controls. Apo-AI levels were also found to be lowest in primary and secondary progressive MS. Furthermore, mice with deficient Apo-AI protein had more severe EAE disease than wild type animals with normal ApoA-I levels^35^. Apo-AI levels declined with age, however levels below 110 mg/dL could be a sign of neurodegenerative disorder readiness. The levels of Apo-A1 decreased with disease progressed. Overexpression of ApoA-I levels has been shown in multiple studies to be effective in delaying the progression of age-related learning and memory impairments in transgenic mouse models^36–38^. Because Apo-A1 is highly expressed in spinal fluid, changes in lipid metabolism can have a negative impact on myelin^39^.

Furthermore, many research on the familial risk of MS have been published. Family studies revealed the prevalence of MS is higher in the families of MS patients than in the general population. The risk of MS among first-degree relatives is seven times higher than in the other populations, according to Nete Munk Nielsen et al. in 2005^40^. H Carton also stated in 1997 that in Flanders the risk of MS is 10–12-fold greater for first-degree and threefold in the second-degree relatives of MS patients^41^. According to a meta-analysis study conducted by Cullen O'Gorman et el, if first-degree relatives, such as father, mother, sister, brother, aunt, and uncle, have MS, the risk of afflicting this disorder is 16.8% higher than in normal other people who do not have a familial risk of MS^42^ . In Iran and around the world, the likelihood getting children with MS from both MS parents are roughly 35–25% higher than in healthy people^43^.

Throughout many research findings, vitamin D levels in patients with MS are significantly lower than in individuals with healthy conditions^43^. However, there were no significant differences in some studies^44^. There are limited and contradicting results revealed for the specificity studies on the degree of vitamin D in MS patients in Iran and the world^45,46^. People with 25(OH)D levels greater than 100 nmol/L (40 ng/mL) had a nearly 62 percent lower risk of getting MS^47^. We measured vitamin D levels in first-degree relatives of untreated RRMS cases as well as RRMS patients, for analyzing Vit D levels and investigating the relationship between vitamin D and MS. The statistical analyses showed a significant reduction of plasma level of vitamin D3 in both RRMS patients and high-risk groups, although, there was no significant association between vitamin D and S100A12 & Apo-A1 levels.

There has been no study of S100A12 biomarker in first-degree relatives of patients with RRMS, according to our findings. In this investigation, we focused on analyzing the levels S100A12 in healthy control, new RRMS cases, and their first-degree family members.

In this study, we indicated that plasma levels of S100A12 were decreased in new untreated RRMS patients compared to the healthy individuals; additionally, the S100A12 plasma levels in the first-degree family (high-risk) was lower than HC group. The results about gender-related shown the plasma level of S100A12 was considerably lower in females than in males in both untreated RRMS and high-risk groups. Plasma levels of Apo-A1 were observed to be considerably lower in the first-degree family than in the HC group. Furthermore, Apo-A1 plasma levels were lower in the new untreated RRMS patients than in the HC group, however this difference was not statistically significant (*P* = 0.379). This non-significant relationship seen in the untreated RRMS patients could be due to the limited number of participants. Also, in untreated RRMS and high-risk groups, females had lower plasma Apo-A1 levels than males. Although there was no significant correlation between the levels of S100A12 and ApoA1 among pairs of RRMS patients and family members, the plasma levels of Apo-A1 & S100A12 were analyzed with age and sex in each group. By our founding, the Apo-A1 plasma levels were lower in the female than in the male among untreated RRMS patients & high-risk groups. Furthermore, concentrations of Apo-A1 and S100A12 plasma showed a positive correlation to age in both RRMS and high-risk groups.

The main goal of starting this research is to use these biomarkers, as well as other biomarkers, as a therapeutic method for early detecting and screening MS in susceptible (high-risk) individuals in the near future. In conclusion, the study of these biomarkers in MS patients is in its early stages, requiring more research and a better understanding of the biomarker's mechanism of action, but based on previous information obtained in other studies, the role of these biomarkers in inflammatory diseases has been well studied and certified. According to this study, this biomarker has been linked to MS as an inflammatory disease and their first-degree relatives.

To explain the relationship between S100A12 or Apo-A1 and familial association, as well as display the mechanism action of S100A12/Apo-A1 as biomarkers in the high-risk group, future studies will require a larger sample size of high-risk and MS patient groups, other biomarkers, and genetic testing such as polymorphism analyses. The results of this paper may assist other researchers in using a variety of biomarkers as treatment strategies for prognosis and screening high-risk MS cases before they develop the disease or while they are already suffering from it.

## Limitations

There were limitations to our study. Because of the patients' initial conditions, the possibility of cooperation in sampling is significantly reduced. Furthermore, due to the multifactorial nature of MS, it is not possible to investigate all of the events that lead to MS.

## Materials and methods

### Patients and controls

The study was performed on 26 females and 9 males as new cases of untreated RRMS patients had no history of consuming immunomodulatory or immunosuppressive medicines, they were referred to the Neurology Clinic and their disease was validated by a neurologist based on laboratory and radiological results. 19 females and 5 males as the healthy control group and 16 females and 10 males joined the study as the first-degree family member of the RRMS patients, consider them as a high-risk group with non-inflammatory neurological diseases. The Expanded Disability Status Scale (EDSS) was used to assess the patients' clinical status. Prior to taking part in the study, all participants voluntarily sign a consent form. All members' names, sexes, ages, dates of birth, heights, and weights were recorded. Also, the age- and sex-matched of healthy controls (HC) were followed.

### Exclusion criteria

At the entry to research, individuals with a history of kidney, liver & heart disease, pregnancy, diabetes type I & П, other neurological and inflammatory diseases, malignancies, fever, or other inflammatory signs were excluded from the study. It's worth noting that no one in the healthy control group had MS or any other neurological or inflammatory condition in their first-degree relatives.

### MRI examination

In two different centers in Shiraz and Isfahan, all MS patients were scanned using a 1.5 Tesla brain magnetic resonance imaging (MRI) with intravenous administration of gadopentate dimeglumine (Gd-DTPA). T1-weighted axial spin-echo images, T2 sequences, and fluid attenuation inversion recovery (FLAIR) images were taken in each patient approximately 10–15 min after injection of 0.1 mmol/kg of (Gd)-DTPA. To confirm the diagnosis of RRMS, all investigations were examined by a single neuroradiologist. If any Gd enhancement was seen on T1-weighted scans, the lesions were classified as active. All brain MRI scans were reviewed by one analyzer who was blinded to the patients and sample findings.

### Plasma sampling

For laboratory testing, 5 cc of venous blood were taken from all participants in the study, samples were poured into ethylenediamine tetra-acetic acid (EDTA) anticoagulant tubes, shaken eight times to avoid blood clotting, then samples were collected on ice in the sterile conditions and transferred to the laboratory for plasma separation, samples were centrifuged for 10 min at 1500 g at 4 °C, the samples aliquoted and preserved at − 70 °C until investigation was performed.

### Measurement of plasma levels of S100A12 & Apo-A1

We measured the plasma levels of S100A12 and Apo-A1 by enzyme-linked immunosorbent assay (ELISA) kit provided by Bosterbio Company (EK1169) & (EK 1456) Pleasanton, respectively. According to the kit instructions, a standard curve in the range of 0–2000 pg/ml was used for S100A12; Plasma samples were diluted (1:100) with the provided diluents.

### Statistical analysis

The mean and standard deviation were used to express all of the data (SD). For analyzing differences between groups of variables with normal distribution, we used one-way analysis of variance (ANOVA) followed by Tukey post-hoc tests. Kruskal–Wallis tests were used to compare groups of variables that were not normally distributed, followed by Mann–Whitney U tests. To analyze relationships between two parameters, Spearman's correlation coefficients were computed. For statistical analysis, the Statistical Package for the Social Sciences (SPSS®) version 16, GraphPad Prism version 8.0.02, and Excel were used. A statistically significant value was defined as *P* 0.05.

### Ethics approval and consent to participate

The Human Research Ethics Committee and Medical Research Governance at Fasa University of Medical Science gave their approval to this investigation (Ethical code number IR.FUMS.REC.1397.016). Prior to being enrolled in the study, all individuals gave their written informed consent. This study followed all relevant rules and regulations, such as the Declaration of Helsinki and the International Council on Good Clinical Practice guidelines. Everyone who took part in the study did so voluntarily.

## Data Availability

Derived data supporting the findings of this study are available from the corresponding author on request.
